# Socioeconomic and Health Determinants of the Prevalence of COVID-19 in a Population of Children with Respiratory Diseases and Symptoms

**DOI:** 10.3390/children11010088

**Published:** 2024-01-11

**Authors:** Agata Wypych-Ślusarska, Karolina Krupa-Kotara, Klaudia Oleksiuk, Joanna Głogowska-Ligus, Jerzy Słowiński, Ewa Niewiadomska

**Affiliations:** 1 Department of Epidemiology, Faculty of Public Health in Bytom, Medical University of Silesia in Katowice, 41-902 Katowice, Poland; awypych@sum.edu.pl (A.W.-Ś.); koleksiuk@sum.edu.pl (K.O.); jglogowska@sum.edu.pl (J.G.-L.); jslowinski@sum.edu.pl (J.S.); 2 Department of Biostatistics, Faculty of Public Health in Bytom, Medical University of Silesia in Katowice, 41-902 Katowice, Poland; eniewiadomska@sum.edu.pl

**Keywords:** asthma, bronchitis, chronic cough, COVID-19, SARS-CoV-2, SES, inequalities

## Abstract

Background: Most epidemiological studies indicate that bronchial asthma is not a risk factor for COVID-19, but previous analyses have not additionally focused on the socioeconomic determinants of SARS-CoV-2 infection in children with asthma, bronchitis, and respiratory symptoms. Aims: This research aimed to investigate the correlation between the socioeconomic status of families and the prevalence of respiratory conditions such as asthma, bronchitis, and respiratory symptoms in children, in addition to exploring their association with the prevalence of COVID-19. The study involved a cross-sectional epidemiological investigation conducted in 2022, encompassing 2454 students from elementary schools in Poland. The parents of the students completed a questionnaire modeled after the International Study on Asthma and Allergies in Childhood (ISAAC). Socioeconomic status (SES) indicators were determined based on parental education, self-reported economic status, and housing conditions. To assess the impact of social factors and health on the occurrence of COVID-19, odds ratios (ORs) were calculated. The findings revealed several COVID-19 risk factors, including higher maternal (OR 2.2; 95%CI: 1.3–3.0) and paternal education (OR 1.9; 95%CI: 1.3–2.4), urban residence (OR 1.7; 95%CI: 1.3–2.1), the presence of mold in residences (OR 1.7; 95%CI: 1.0–2.3), bronchitis (OR 1.5; 95%CI: 1.2–2.0), and chronic cough (OR 1.8; 95%CI: 1.3–2.4). Further analysis, stratifying children based on their baseline health status (i.e., presence or absence of asthma, bronchitis, and chronic cough), indicated that higher parental education increased the risk of COVID-19 solely for children without pre-existing conditions. The occurrence of SARS-CoV-2 infections was found to be notably associated with mold exposure in children who did not have bronchial asthma. Rigorous multivariate analyses substantiated the collective impact of factors such as residential environment, the existence of mold and moisture, and a history of bronchitis. This study’s conclusions highlight a higher frequency of SARS-CoV-2 infections in cases where bronchitis had been diagnosed previously and chronic cough was prevalent. Interestingly, the initially hypothesized higher prevalence of COVID-19 among children with bronchial asthma did not receive confirmation in our findings. This study highlights the importance of urban residence, exposure to mold or dampness, and higher parental education in the incidence of COVID-19. Higher parental education was a significant factor in increasing the risk of COVID-19 among children without bronchitis, chronic cough, and asthma.

## 1. Introduction

The global impact of the COVID-19 pandemic, attributed to the novel SARS-CoV-2 virus, has heightened global awareness regarding the intricate interplay between infectious diseases and their interconnections with chronic illnesses, as well as the broader influence of social and environmental factors. Since its outbreak, many scientific papers have been written on COVID-19, initially focusing on understanding the mechanisms of the disease and its determinants and risk factors. In the final stages of the pandemic and the post-pandemic period, the scientific community’s efforts have focused on analyzing the links between COVID-19 and chronic diseases, mental health, and the effects resulting from the pandemic [1,2]. 

The identification of factors contributing to elevated risk and a more severe progression of COVID-19 has become increasingly elucidated, with chronic diseases such as diabetes, cardiovascular ailments, cancer, and certain respiratory conditions featuring prominently in the list [3]. In the context of bronchial asthma, there has been apprehension regarding its potential role as a contributing factor to an elevated risk and a more severe trajectory of COVID-19 [4]. Notably, the Pediatric Section of the European Academy of Allergy and Clinical Immunology (EAACI), in a formal statement, suggested that individuals with asthma, particularly those with severe or poorly controlled cases, should be categorized as having an increased susceptibility to a more severe course of COVID-19. It is crucial to note, however, that this standpoint was primarily grounded in logical reasoning rather than robust scientific evidence [5]. Nevertheless, the scrutiny of adult-focused investigations failed to establish asthma or even chronic obstructive pulmonary disease (COPD) as significant risk factors, attributing this finding to their relatively lower representation among the reported comorbidities associated with COVID-19 [6]. Most studies indicating an association between asthma and COVID-19 have focused on adults, even though, according to multicenter epidemiological data, SARS-CoV-2 infections among children accounted for about 16% of all laboratory-confirmed cases [7,8]. Children generally have a milder course of SARS-CoV-2 infection and have generally not been the focus of analyses in this regard [7,8,9]. Although most studies indicate that there is no association between asthma and the severity of COVID-19 in the pediatric population [4,10,11], some reports confirm the association of this disease with the course of SARS-CoV-2 infections [12,13,14]. For example, within a cohort of British children aged 5–17 years who had experienced at least one hospitalization for asthma, the observed risk of hospitalization for COVID-19 was six times greater compared to children without asthma [13]. Moreover, recent findings from a retrospective study involving individuals below the age of 21 years indicated a heightened risk of hospitalization due to COVID-19 in the presence of asthma [12]. Analyses of COVID-19 risk factors also point to its socioeconomic and environmental determinants. Typically, studies focus on framing these factors in macrosocial or macroeconomic terms [15,16]. Research data demonstrate an association between urban residence (higher population density than in rural areas), having ethnic minority status, living in poor neighborhoods, or lack of or difficult access to medical care and the risk of COVID-19. Still, few studies have focused on individual socioeconomic risk factors. For asthma and respiratory symptoms, such analyses have become commonplace. 

Given these contextual considerations, it appeared intriguing to conduct an examination aimed at elucidating the interplay between the socioeconomic status of families, the prevalence of asthma, bronchitis, and respiratory symptoms in children, and the overall prevalence of COVID-19. However, to the best of our knowledge, there have been no analyses indicating the socioeconomic determinants of COVID-19 in a population of children with asthma, bronchitis, and respiratory symptoms. Asthma is additionally influenced by socioeconomic and environmental factors, elements that hold non-negligible relevance in the context of COVID-19’s prevalence. Hence, this inquiry sought to address the following research questions:

1. Are asthma, bronchitis, or respiratory symptoms and socioeconomic factors risk factors for COVID-19?
2. Do selected socioeconomic factors differentiate the prevalence of COVID-19 among children with respiratory diseases and symptoms?

## 2. Materials and Methods

### 2.1. Study Group Attributes

In the initial six months of 2022, a cross-sectional investigation was carried out encompassing a cohort of 2454 children aged between 5 and 15 years residing in Poland. For statistical analyses, the respondents were divided according to the following age groups: 5–8 years (*n* = 637; 23.1%), 9–12 years (*n* = 1237; 50.7%), and 13–15 years (*n* = 563; 23.1). Data on the children’s health status were collected through a questionnaire that was completed by the children’s parents.

### 2.2. Eligibility Criteria

This study employed a cluster selection methodology, wherein 16 localities, representing municipalities or cities with county rights across diverse demographic levels, were randomly chosen from each of the 16 provinces in Poland. Invitations to partake in the study were extended to school principals in the selected localities. Utilizing data from the Central Statistical Office about the population’s state and structure, a minimum sample size commensurate with the population of children aged 5–15 in Poland was computed (N = 4,326,314). With a confidence level of 95%, a fraction size of 0.5, and a maximum error of 5%, the minimum sample size for children with COVID-19 was determined to be 385. Assuming a prevalence rate of 16% among the population [8], the initial sample size was set at 2406. Considering a 2% margin of error on the questionnaire’s accuracy, the final sample size was established not to exceed *n* = 2455. The primary inclusion criterion was the parental consent for the children to participate in the study, indicated through the completion of the questionnaire. Participation was entirely voluntary and anonymous, adhering to the principles of the Declaration of Helsinki. The study design, as per the Act of 5 December 1996 on the professions of physician and dentist, was deemed not to be a medical experiment and, therefore, did not necessitate approval from the Bioethics Committee of the Silesian Medical University in Katowice (opinion no. PCN/CBN/0052/KB/190/22, 4 October 2022). Additionally, the data collection was premised on an anonymous questionnaire, for which parents provided voluntary consent. The distribution of questionnaires directly to parents of school-aged children ensured that the absence of a completed questionnaire signified a lack of consent to participate in the study.

### 2.3. Research Tool

The data collection instrument utilized in this study took the form of an anonymized questionnaire, drawing inspiration from the structure employed in the International Study of Asthma and Allergies in Childhood (ISAAC). The survey administration was conducted through computer-assisted web interviewing (CAWI), utilizing the available MS 365 Forms software (Microsoft Cooperation, Redmond, WA, USA, edition 18.2306.1061.0) tools for efficient and streamlined data collection. For the aims of this study, the criteria for identifying bronchial asthma and bronchitis were contingent upon a positive response to inquiries regarding the historical diagnosis of these conditions by a qualified medical professional. This approach ensured a standardized and clear definition for the assessment of bronchial asthma and bronchitis within the research framework. The question on asthma was “Has a doctor ever diagnosed your child with bronchial asthma?”, and the question on bronchitis was “Has a doctor ever diagnosed your child with bronchitis?” Also, data on the prevalence of respiratory symptoms in the children were obtained from information provided by the parents in a questionnaire survey. Chronic cough was defined as a cough that lasts at least three months outside of cold periods. The prevalence of SARS-CoV-2 infection was calculated based on the parents’ positive response to the question “Has the child undergone SARS-CoV-2 coronavirus infection, confirmed by a positive RT- PCR test or antigen test (for self-testing)?”, with three possible answers: “Yes, confirmed by RT- PCR test”, “Yes, confirmed by antigen test”, or “No”. The first two answers provided the basis for qualifying the child as having had COVID-19, while the last answer ruled out this diagnosis.

Socioeconomic variables included the following:

- The education of the child’s father and mother, defined as low (in the case of primary or vocational education), middle (in the case of secondary education), or high (in the case of higher education);
- Self-assessment of the material situation (good, average, or bad);
- Whether the child has siblings (yes or no);
- The existence of indications of mold and dampness within the dwelling over the preceding 24 months (current, present but mitigated, or never present).

All data about the residential environment of the children were acquired through the responses provided in the questionnaire. Parental completion of the questionnaire was the method employed for data collection. To safeguard the privacy and anonymity of the participants, all information was encoded using designated symbols, in adherence to the provisions outlined in the Act of 29 August 1997 concerning the protection of personal data (Journal of Laws 1997 No. 133 item 883).

### 2.4. Statistical Analyses

Statistical calculations were performed using the STATISTICA 13.0 program, Stat Soft Poland. Data are presented in numeric-percentage notation–*n* (%). To assess the relationships between qualitative variables, the chi-squared test was used. The prevalence of COVID-19 was also compared according to socioeconomic factors for all children studied, as well as for children with symptoms and/or diseases for which analyses showed an association with the prevalence of COVID-19. 

The impact of socioeconomic status (SES) on the incidence of COVID-19 was examined through univariate logistic regression. Only variables demonstrating a statistically significant relationship in the preliminary analyses were incorporated. Unadjusted models, featuring crude odds ratios, were employed with individual independent variables such as habitation, the presence of mold or dampness in the dwelling, and parental education status. Furthermore, adjusted odds ratios, accounting for sex and age variables, were calculated along with 95% confidence intervals (OR (%CI)). Lastly, multivariate logistic regression models were employed to discern the distinct impact of the investigated factors on the occurrence of COVID-19. Statistical significance was established at *p* ≤ 0.05. 

## 3. Results

A comprehensive examination was conducted on a cohort of 2454 children, comprising 1299 boys (52.9%) and 1155 girls (47.1%), with ages ranging from 5 to 15 years (mean age 10.4 ± 2.3 years). In 423 children (17.4%), SARS-CoV-2 coronavirus infection was confirmed by a positive RT- PCR or antigen test. 

Most of the children resided in cities (*n* = 1617, 65.9%), while 837 were rural residents (34.1%). More than half of the surveyed mothers declared a higher education (*n* = 1400, 57.9%), 30.4% (*n* = 734) of them had secondary education, and 11.7% (*n* = 284) had primary or vocational education. The educational structure of the surveyed fathers was as follows: 24.6% (*n* = 561) of fathers had primary or vocational education, 35.5% (*n* = 811) had secondary education, and 39.9% (*n* = 911) had higher education. About ¾ of the children had siblings (*n* = 1926, 79.7%) and came from well-off homes (*n* = 1650, 68.3%). In the case of 727 children (30.1%), the parents declared an average material situation, while the parents of 40 children (1.6%) declared a bad one. The occurrence of mold or moisture within the residence was reported by 156 respondents (6.5%), while 425 parents (17.7%) attested to their successful removal, and 1819 parents (75.8%) confirmed their absence. Most parents rated their child’s health as very good (*n* = 2256; 92.0%), 179 as average (7.3%), and 16 as poor (0.7%). Bronchial asthma affected 372 children (15.2%), while bronchitis affected 465 children (18.9%). The most reported respiratory symptoms were wheezing ever (*n* = 635; 26.0%) and dyspnea ever (*n* = 368; 15.0%).

SARS-CoV-2 virus infection was confirmed statistically significantly more often in children living in cities than in villages. Most respiratory symptoms were more common in younger children (5–8 years). Statistically significant findings revealed a higher likelihood among boys of experiencing chronic cough, wheezing ever and in the last 12 months, and dyspnea ever, as well as instances of bronchitis and asthma, as outlined in Table 1.

**Figure 1:**
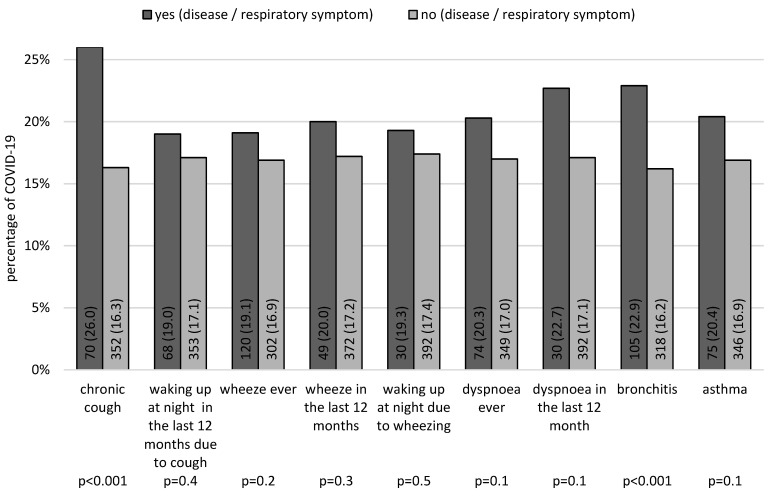
Prevalence of COVID-19 among children without or with a diagnosis of asthma, bronchitis, and/or respiratory symptoms (data presented as numbers and percentages, *n* (%); *p*-values—the results of the chi-squared tests).

Analysis of the data showed that COVID-19 was significantly more common in children with chronic cough and bronchitis (Figure 1). 

**Figure 2:**
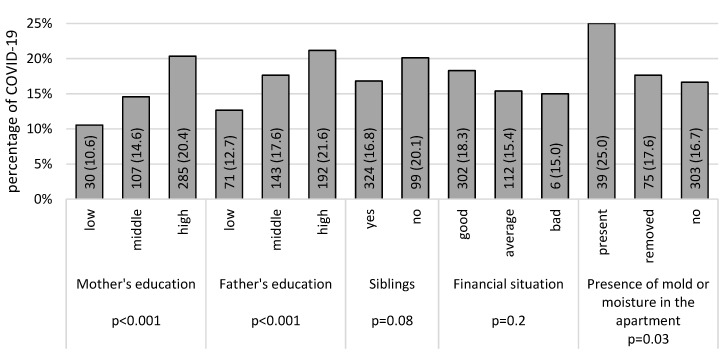
Prevalence of COVID-19 according to socioeconomic conditions (data presented as numbers and percentages, *n* (%); *p*-values—the results of the chi-squared tests).

COVID-19 was significantly more common in children whose parents had a college education and who lived in homes with traces of mold or moisture (Figure 2). A statistically significant correlation with COVID-19 incidence was not confirmed for children whose parents declared a good financial situation. 

Logistic regression analysis confirmed the importance of selected factors in the incidence of COVID-19 in the entire group of children studied. The risk of COVID-19 was higher in children living in the city than those living in the countryside (OR = 1.7; 95%CI: 1.3–2.1), in those whose parents had a university education compared to primary education (mother: OR = 2.2; 95%CI: 1.3–3.0; father OR = 1.9; 95%CI: 1.3–2.4), and in those whose homes had traces of dampness or mold as opposed to none (OR = 1.7; 95%CI: 1.0–2.3). In addition, bronchitis (OR = 1.5; 95%CI: 1.2–2.0) and chronic cough increased the risk of COVID-19 (Table 2). The adjusted odds ratios by sex and age remained similar relative to the unadjusted odds ratios. 

In light of the established associations, our inquiry aimed to ascertain whether the incidence of COVID-19 in children with asthma, bronchitis, and chronic cough is contingent upon socioeconomic conditions, as delineated in Table 3. Regardless of the presence of diseases or respiratory symptoms, the analyses confirmed an increase in the rate of COVID-19 infection among children as their parents’ education increased, when mold or dampness was present in the home, and when living in an urban area. However, only for children without respiratory diseases or symptoms was parental education significant for the incidence of COVID-19, but it should be noted that COVID-19’s incidence was higher in children who presented with respiratory diseases or symptoms.

Multivariate models were considered to isolate the significant effects of the studied factors on the occurrence of COVID-19. The results are presented in Table 4. It was observed that cities as a place of residence significantly increased the risk of COVID-19 when the possibility of bronchitis or asthma was included in the model (OR = 1.7; 95%CI: 1.3–2.1 and OR = 2.4; 95%CI: 1.3–4.6, respectively). In addition, in all of the models presented, the risk of COVID-19 was significantly higher with mold or dampness. Among other respiratory illnesses or symptoms, only bronchitis significantly increased the risk of SARS-CoV-2 infection (OR = 1.5; 95%CI: 1.2–2.0).

## 4. Discussion

The study that we conducted aimed to determine the relationship between the socioeconomic situation of families, the prevalence of asthma, bronchitis, and respiratory symptoms in children, and the incidence of COVID-19. This is the first study of its kind conducted in Poland and one of the few indicating an association between health status, individual socioeconomic factors, and the incidence of COVID-19 in a group of school-aged children. Previous studies focusing on the determinants of the social environment and economic situation have been conducted concerning the structural dimension, taking into account the macrosocial and macroeconomic situation [15,16]. These studies made it possible to identify significant variations in the numbers of infected and deceased in connection with socioeconomic factors, demographics, and access to medical care at both regional and national levels [17,18]. However, these analyses were mostly based on an ecological survey model and did not identify individual risk factors. Meanwhile, the ongoing debate as to whether asthma is a risk or protective factor for COVID-19 prompts a broader perspective on the issue. It seems indispensable to include in the analyses the determinants of the social and economic environment, which are known and documented risk factors for bronchial asthma and influence the prevalence of respiratory symptoms in children. Therefore, the determinants of the home environment may also be important factors influencing the variation in the prevalence of COVID-19 in the studied group of children. 

The results of this study revealed the prevalence of bronchial asthma in the analyzed group to be 15.2%, while 17.4% of the children were affected by COVID-19. SARS-CoV-2 virus infection was more often reported by the parents of children living in cities than by those living in villages. This observation is consistent with the results of other studies and may be explained by differences in population density and social patterns of daily activity [15]. In addition, COVID-19 is statistically significantly more common in children with chronic cough and bronchitis. Also, a higher prevalence of SARS-CoV-2 infection was observed in bronchial asthma, but the differences with respect to the group of children without asthma were no longer statistically significant. At the time of the pandemic’s outbreak, it was feared that asthma might be a risk factor for severe COVID-19, as some studies have confirmed [19]. The results of a cohort study conducted in the UK indicated that asthma was a risk for more frequent hospitalization due to COVID-19 [19]. However, it should be noted that the cohort studied included adults aged 48–85 years, so these observations cannot be taken as a reference for the situation of the pediatric population. Subsequent studies have no longer confirmed this association [20,21]. Moreover, recent analyses suggest a protective effect of bronchial asthma against COVID-19 infections [21]. An important role is played by the cytokine IL-13, which downregulates the expression of the viral receptor ACE2, contributing to a reduction in the numbers of viral particles inside cells and limiting viral replication. In addition, IL-13 enhances ion transport and intensifies antiviral processes, which are crucial in airway immune defense [21]. 

In contrast, our own studies have shown a higher prevalence of COVID-19 when a child has a coexisting chronic cough or bronchitis. Chronic cough is a well-known symptom of many diseases in children, such as bronchial asthma, bronchitis, cystic fibrosis, lung disease, and COVID-19 [22]. Epidemiological data indicate that this symptom can affect up to 22% of children [22,23]. In this study, however, the parents of the children were not asked about the prevalence of all diseases that may be accompanied by chronic cough. They were limited only to asthma, bronchitis, and COVID-19. Therefore, it cannot be ruled out that the symptom of chronic cough was associated with the presence of other diseases or frequent infections that are characteristic of childhood. On the other hand, some studies indicate a more severe course of COVID-19 in children with comorbidities and higher rates of inflammation [14,23,24]. An analysis of data from one center admitting children with COVID-19 in Poland showed that children with comorbidities, chronic cough, and dyspnea were more likely to require oxygen therapy and were more likely to be admitted to the intensive care unit [25]. 

Risk factors for the onset and severity of COVID-19 may also depend on socioeconomic conditions [26]. Studies indicate an association between low economic status, living in marginalized communities, poor-quality housing or precarious work, and a higher incidence of COVID-19 [26,27,28,29,30]. As mentioned above, most studies focus on social and economic differences in the incidence and course of COVID-19 at the macro level. However, it is known that the population dimension intersects with the individual dimension. Living in poor neighborhoods can be associated with economic constraints and the inability to change one’s area of residence. The economic situation frequently affects the quality of housing and the presence of certain factors that increase the risk of allergic or infectious diseases. Studies show that multidimensional poverty also influences lifestyles that promote the onset of chronic, non-communicable diseases [26]. 

The results of our study show the influence of higher education for the mother and father of the child, as well as of the presence of traces of mold and dampness in the residence, on the incidence of COVID-19. However, to explain the former observation, we must conclude with a hypothesis regarding the association of occupation with a higher incidence of SARS-CoV-2 infections. However, this hypothesis is not verifiable through additional analyses of the present study, since the protocol did not include questions about the parents’ occupational activity. It is possible, however, that higher levels of education were related to occupation, perhaps even in the services that were involved in the fight against the pandemic. For example, the results of an analysis of COVID-19 incidence data among healthcare workers in Poland revealed a higher prevalence of infection in the study group than in the general population [31]. However, an attempt to explain the observation indicating a relationship between education and the incidence of COVID-19 based on the results of our study will remain in the realm of speculation and, thus, will not have the hallmarks of scientific inference. This is therefore a limitation of the present study, but at the same time a suggestion for expanding the range of significant demographic variables in similar such analyses. Moreover, the present observation differs from the results of studies indicating that it is lower levels of education that are associated with a higher risk of severe COVID-19 [32,33]. 

However, an unquestionable observation is the statistically significant relationship between the presence of mold and moisture in children’s residences and the incidence of COVID-19. First, during the lockdown period, all previous work, school, and leisure activities were confined to the space of the residence (i.e., home or apartment). Indoor air quality therefore gained in importance. The results of epidemiological studies indicate an association between outdoor as well as indoor air pollution and higher rates of SARS-CoV-2 infection [34,35]. In addition, exposure to moisture, molds, and fungi is a known and confirmed risk factor for many allergic and respiratory diseases [36,37,38]. Poor indoor air quality is often associated with lower socioeconomic status. This association can be translated into simple links suggesting that the inability to meet all needs leads to deterioration in the quality of inhabited housing, which promotes increased exposure to allergens and fungi and, ultimately, a higher incidence of disease. However, the results of our study are no longer so conclusive, as they indicate a higher incidence of COVID-19 in the group of children whose parents declared a good financial situation. Admittedly, the differences between the groups were not statistically significant, so they may be a matter of chance. It is possible, however, that respondents’ self-assessment of their economic situation is not exactly a reliable indicator. Often, however, this remains the only way to determine the economic status of the respondents, since direct questions about the amount of income are among the sensitive ones and are often ignored by respondents. 

The logistic regression results of this study confirmed a higher risk of COVID-19 in the entire study group of children for the presence of chronic cough, ever-diagnosed bronchitis, higher parental education, and mold exposure in the residence. Interestingly, analyses differentiating children by health status for the presence or absence of asthma, bronchitis, and chronic cough showed the significance of these risk factors only for healthy children. Higher parental education was associated with a higher prevalence of COVID-19 in the group of children without asthma, bronchitis, or chronic cough, but it no longer affected the frequency of SARS-CoV-2 infection for children with these diseases. Compared to the group of children with asthma, children without the disease were more frequently diagnosed with COVID-19 when traces of mold were present in their homes. This observation is important because it may highlight the importance of an individual’s perception of risk in the actions they take that may reduce the risk of a particular disease. Risk perception varies with socioeconomic situation but can also depend on health status [39]. The occurrence of a disease or the severity of its symptoms may prompt an individual to eliminate or reduce exposure to known risk factors. Therefore, it cannot be ruled out that in the case of a child’s diagnosis and the presence of a chronic cough, parents’ concern for the quality of the home environment and the elimination of allergens is greater than that of the parents of healthy children. This result further emphasizes the importance of mold exposure in the incidence of COVID-19. 

### Strengths and Limitations

The strength of the present study is that it analyzed the relationships between selected socioeconomic factors, children’s health status, and the prevalence of COVID-19. The study that we conducted, investigating a group of children with asthma, bronchitis, and chronic cough with respect to the analyzed socioeconomic determinants and SARS-CoV-2 infections, is a novel approach to the topic and also indicates the need for education about known risk factors for respiratory diseases in populations free of these diseases. In addition, questions from an international standardized questionnaire used in the ISAAC study were used to assess the prevalence of respiratory illness and symptoms. What is important in this study is its holistic approach to health, which considers not only symptoms, but also conditions, and attempts to explain the variation in COVID-19’s prevalence in different subgroups of the studied children. 

The main limitation of the present study, however, was the survey model adopted. The cross-sectional survey is an appropriate model for a preliminary assessment of health status and an indication of possible relationships between variables. This is also how the analyses conducted in this study should be interpreted. However, the observed relationships should not be interpreted as cause-and-effect relationships. The reliance on self-reported data, particularly regarding respiratory conditions and housing conditions, may introduce recall bias and subjective interpretation. Objective clinical assessments and environmental evaluations could enhance the accuracy of the information gathered. This study’s focus on elementary school students in Poland may limit the generalizability of the findings to other age groups or geographical regions. Diverse demographic and cultural factors might influence the observed relationships differently in various populations. The completion of questionnaires by parents introduces the potential for response bias, as parents might underreport or overreport certain information based on perceptions or social desirability. The inclusion of additional objective measures could enhance the validity of the findings. While this study considered parental education, self-reported economic status, and housing conditions as indicators of socioeconomic status (SES), other relevant factors, such as household income or employment status, were not included. A more comprehensive SES assessment could provide a nuanced understanding of its impact. Despite statistical adjustments, the presence of unmeasured confounding factors could influence the observed associations. Variables like access to healthcare, individual health behaviors, and community-level factors were not explicitly addressed in the analysis. This study did not delve into the nuances of COVID-19 testing protocols or variations in testing accessibility, which might impact the reported prevalence rates. Future research could explore these aspects to refine the accuracy of COVID-19 incidence estimates.

Despite these limitations, this study provides valuable insights into the complex interplay between socioeconomic factors, respiratory conditions, and the prevalence of COVID-19 among children. Further research employing diverse methodologies and addressing these limitations could enhance the depth and applicability of our understanding in this area. Most of the limitations associated with the study model used here have already been discussed in the Discussion. However, the results of the present study are a good starting point for hypothesizing and seeking answers in models for more complex observational studies. 

## 5. Conclusions

The prevalence of COVID-19 in the study group of children did not depend on the presence of bronchial asthma, while SARS-CoV-2 infections were more common with any diagnosis of bronchitis ever and the presence of chronic cough. 

Residing in urban areas and/or the existence of mold or dampness within the living quarters emerged as risk factors for COVID-19.

In an analysis differentiating children by baseline health status (i.e., presence or absence of asthma, bronchitis, and chronic cough), higher parental education appeared to increase the risk of COVID-19 only for children without baseline diseases. 

Educational activities on basic risk factors for respiratory and allergic diseases should also be carried out in the general population to sensitize them to the possible impact of these conditions on the risk of diseases during epidemic periods. 

## Figures and Tables

**Table 1:** Prevalence of diseases and respiratory symptoms according to demographic variables.

Disease/Respiratory Symptom	Total	Habitation		*p*-Value	Sex		*p*-Value	Age			*p*-Value
		Town *n* = 1617	Village *n* = 837		Boy *n* = 1299	Girl *n* = 1155		5–8 *n* = 637	9–12 *n* = 1237	13–15 *n* = 563	
Chronic cough	272 (11.1)	193 (11.9)	79 (9.5)	0.06	167 (12.9)	105 (9.1)	0.002	88 (13.8)	129 (10.4)	51 (9.1)	0.02
Waking up at night in the last 12 months due to cough	363 (14.8)	236 (14.6)	127 (15.2)	0.7	202 (15.6)	161 (19.5)	0.2	114 (17.9)	187 (15.1)	59 (7.5)	0.001
Wheezing ever	635 (26.0)	421 (26.2)	214 (25.7)	0.8	389 (30.1)	246 (21.4)	<0.001	168 (26.5)	342 (27.8)	115 (20.6)	0.004
Wheezing in the last 12 months	246 (10.1)	162 (10.1)	84 (10.1)	0.9	148 (11.5)	98 (8.5)	0.01	89 (14.1)	116 (9.5)	37 (6.6)	<0.001
Waking up at night due to wheezing	157 (6.4)	107 (6.6)	50 (6.0)	0.5	87 (6.7)	70 (6.1)	0.5	64 (10.1)	65 (5.3)	24 (4.3)	<0.001
Dyspnea ever	368 (15.0)	248 (15.4)	120 (14.3)	0.5	227 (17.5)	141 (12.2)	<0.001	91 (14.3)	191 (15.5)	83 (14.8)	0.7
Dyspnea in the last 12 months	133 (5.4)	94 (5.8)	39 (4.7)	0.2	80 (6.2)	53 (4.6)	0.08	43 (6.8)	64 (5.2)	23 (4.1)	0.1
Bronchitis	465 (18.9)	314 (19.4)	151 (18.0)	0.4	300 (23.1)	165 (14.3)	<0.001	127 (19.9)	239 (19.3)	95 (16.9)	0.3
Asthma	372 (15.2)	250 (15.5)	122 (14.7)	0.6	229 (17.7)	143 (12.5)	<0.001	94 (14.8)	192 (15.6)	84 (15.0)	0.8
COVID-19	423 (17.4)	316 (19.7)	107 (12.9)	<0.001	227 (17.7)	196 (17.1)	0.7	98 (15.6)	223 (18.2)	100 (18.0)	0.3

Data presented as numbers and percentages, *n* (%); *p*-values—the results of the chi-squared tests.

**Table 2:** Crude and adjusted odds ratios (ORs) and 95% confidence intervals (95%CIs) related to determinants of COVID-19.

Determinants of COVID-19 (* Reference Group)	OR (95%CI) Crude	*p*-Value	OR (95%CI) Adjusted by Sex and Age	*p*-Value
Place of residence				
Village/city *	1.7 (1.3–2.1)	<0.001	1.7 (1.3–2.1)	<0.001
Mother’s education				
Low */middle	1.4 (0.8–2.1)	0.09	1.5 (1.0–2.3)	0.06
Middle */high	1.5 (1.1–1.9)	<0.001	1.5 (1.2–1.9)	0.001
Low */high	2.2 (1.3–3.0)	<0.001	2.3 (1.6–3.5)	<0.001
Father’s education				
Low */middle	1.5 (1.0–1.9)	0.01	1.5 (1.1–2.0)	0.01
Middle */high	1.3 (1.0–1.6)	0.04	1.3 (1.0–1.7)	0.03
Low */high	1.9 (1.3–2.4)	<0.001	1.9 (1.4–2.6)	<0.001
Presence of mold or moisture in the apartment				
None */removed	1.1 (0.8–1.4)	0.6	1.0 (0.8–1.4)	0.7
Removed */present	1.6 (0.9–2.2)	0.05	1.6 (1.0–2.5)	0.02
None */present	1.7 (1.0–2.3)	0.008	1.7 (1.2–2.5)	0.007
Bronchitis				
No/yes *	1.5 (1.2–2.0)	<0.001	1.5 (1.2–2.0)	<0.001
Chronic cough				
No/yes *	1.8 (1.3–2.4)	<0.001	1.8 (1.4–2.4)	<0.001
Asthma				
No/yes *	1.3 (0.9–1.6)	0.1	1.2 (0.9–1.6)	0.1

Results of unadjusted and adjusted logistic regression models presented as odds ratios with 95% confidence intervals—OR (95%CI).

**Table 3:** Prevalence of COVID-19 in children without or with diagnosed asthma, bronchitis, or chronic cough according to socioeconomic factors.

Disease/ Respiratory Symptom		Yes			No		
Asthma		COVID-19 Yes	COVID-19 No	*p*-Value	COVID-19 Yes	COVID-19 No	*p*-Value
Mother’s education	Low	5 (12.2)	36 (87.8)	0.8	24 (10.1)	214 (89.9)	<0.001
	Middle	18 (16.1)	95 (83.9)		89 (14.3)	531 (85.7)	
	High	51 (24.2)	160 (75.8)		233 (19.7)	951 (80.3)	
Father’s education	Low	13 (15.1)	73 (84.9)	0.2	57 (12.1)	412 (87.9)	<0.001
	Middle	27 (19.6)	111 (80.4)		116 (17.3)	553 (82.7)	
	High	32 (25.4)	94 (74.6)		164 (20.9)	619 (79.1)	
Presence of mold or moisture	Current	8 (27.6)	21 (12.4)	0.6	31 (24.4)	96 (75.6)	0.05
	Removed	12 (18.2)	54 (81.8)		62 (17.5)	292 (82.5)	
	No	55 (20.4)	215 (79.6)		247 (16.0)	1295 (84.0)	
Place of residence	Village	107 (12.9)	720 (87.1)	<0.001	88 (12.5)	613 (87.5)	<0.001
	City	316 (19.7)	1283 (80.3)		258 (19.2)	1087 (18.8)	
Chronic cough		COVID-19 Yes	COVID-19 No	*p*-Value	COVID-19 Yes	COVID-19 No	*p*-Value
Mother’s education	Low	9 (16.4)	46 (83.6)	0.2	21 (9.2)	208 (90.8)	<0.001
	Middle	22 (26.2)	62 (73.8)		85 (13.1)	563 (86.9)	
	High	38 (29.7)	90 (70.3)		246 (19.4)	1024 (80.6)	
Father’s education	Low	16 (21.0)	60 (78.9)	0.2	55 (11.3)	430 (88.7)	<0.001
	Middle	26 (26.5)	72 (73.5)		116 (16.3)	594 (83.7)	
	High	26 (33.3)	52 (66.7)		171 (20.5)	661 (79.5)	
Presence of mold or moisture	Current	10 (37.0)	17 (63.0)	0.3	29 (22.5)	100 (77.5)	0.1
	Removed	12 (24.0)	38 (76.0)		63 (16.8)	312 (83.2)	
	No	44 (23.5)	143 (76.5)		258 (15.9)	1370 (84.1)	
Place of residence	Village	12 (15.2)	67 (84.8)	0.009	94 (12.6)	652 (87.4)	<0.001
	City	58 (30.5)	132 (69.5)		258 (18.3)	1149 (81.7)	
Bronchitis		COVID-19 Yes	COVID-19 No	*p*-Value	COVID-19 Yes	COVID-19 No	*p*-Value
Mother’s education	Low	9 (16.1)	47 (83.9)	0.08	21 (9.2)	207 (90.8)	<0.001
	Middle	25 (18.5)	110 (81.5)		82 (13.7)	517 (86.3)	
	High	70 (26.6)	193 (73.4)		215 (18.9)	922 (81.1)	
Father’s education	Low	17 (15.6)	93 (84.5)	0.2	57 (12.0)	397 (88.0)	0.001
	Middle	36 (22.2)	126 (77.8)		104 (16.5)	542 (83.5)	
	High	49 (29.9)	115 (70.1)		148 (19.8)	599 (80.2)	
Presence of mold or moisture	Current	14 (35.9)	25 (64.1)	0.1	25 (21.4)	92 (78.6)	0.2
	Removed	18 (20.9)	68 (79.1)		57 (16.8)	282 (83.2)	
	No	70 (21.5)	256 (78.5)		233 (15.6)	1260 (84.4)	
Place of residence	Village	27 (18.1)	122 (82.8)	0.08	80 (11.8)	598 (88.2)	<0.001
	City	78 (25.3)	231 (74.7)		238 (18.4)	1052 (81.6)	

**Table 4:** Results of multivariate analyses for the prevalence of COVID-19 in relation to socioeconomic and health factors.

Determinants of COVID-19 (* Reference Group)	Model 1 Bronchitis OR (95%CI)	*p*-Value	Model 2 Chronic Cough OR (95%CI)	*p*-Value	Model 3 Asthma OR (95%CI)	*p*-Value
Place of residence						
Village/city *	1.7 (1.3–2.1)	<0.001	1.1 (0.7–1.7)	0.6	2.4 (1.3–4.6)	<0.001
Mother’s education						
Low/middle *	1.4 (0.8–2.1)	0.2	1.4 (0.9–2.5)	0.1	1.4 (0.8–2.4)	0.2
Middle */high	1.5 (1.1–1.9)	0.8	1.1 (0.6–2.0)	0.8	1.1 (0.6–2.0)	0.8
Low */high	1.7 (0.8–3.7)	0.1	1.8 (0.8–3.9)	0.1	1.7 (0.8–3.7)	0.2
Father’s education						
Low */middle	1.3 (0.8–2.0)	0.2	1.2 (0.8–1.9)	0.3	1.3 (0.8–2.0)	0.7
Middle */high	1.3 (0.9–1.6)	0.2	1.5 (0.8–2.5)	0.2	1.4 (0.8–2.5)	0.8
Low */high	1.7 (1.0–2.8)	0.06	1.7 (1.0–2.9)	0.05	1.7 (1.0–2.8)	0.06
Presence of mold or moisture in the apartment						
None */removed	1.1 (0.7–1.9)	0.6	1.2 (0.7–2.0)	0.5	1.2 (0.7–1.9)	0.6
Removed */present	1.6 (0.9–2.2)	0.1	1.5 (0.9–2.6)	0.1	1.5 (0.9–2.6)	0.1
None */present	2.8 (1.5–4.9)	<0.001	2.8 (1.7–5.0)	<0.001	1.8 (1.2–3.7)	<0.001
Disease/symptom						
No/yes *	1.5 (1.2–2.0)	<0.001	1.6 (0.9–2.8)	0.1	1.1 (0.6–2.0)	0.7

Results of adjusted logistic regression models presented as odds ratios with 95% confidence intervals—OR (95%CI).

## Data Availability

The data presented in this study are available in article.
